# Glycosylated Nanoparticles for Cancer-Targeted Drug Delivery

**DOI:** 10.3389/fonc.2020.605037

**Published:** 2020-11-30

**Authors:** Sergio Andrés Torres-Pérez, Cindy Estefani Torres-Pérez, Martha Pedraza-Escalona, Sonia Mayra Pérez-Tapia, Eva Ramón-Gallegos

**Affiliations:** ^1^ Laboratorio de Citopatología Ambiental, Departamento de Morfología, Escuela Nacional de Ciencias Biológicas, Instituto Politécnico Nacional, Campus Zacatenco, Mexico City, Mexico; ^2^ CONACYT-UDIBI-ENCB-Instituto Politécnico Nacional, Unidad Profesional Lázaro Cárdenas, Mexico City, Mexico; ^3^ Unidad de Desarrollo e Investigación en Bioprocesos (UDIBI), Escuela Nacional de Ciencias Biológicas, Instituto Politécnico Nacional, Mexico City, Mexico

**Keywords:** drug delivery, glycoconjugates, glycosylated nanoparticles, glycodendrimers, cancer therapy, glycopolymers

## Abstract

Nanoparticles (NPs) are novel platforms that can carry both cancer-targeting molecules and drugs to avoid severe side effects due to nonspecific drug delivery in standard chemotherapy treatments. Cancer cells are characterized by abnormal membranes, metabolic changes, the presence of lectin receptors, glucose transporters (GLUT) overexpression, and glycosylation of immune receptors of programmed death on cell surfaces. These characteristics have led to the development of several strategies for cancer therapy, including a large number of carbohydrate-modified NPs, which have become desirable for use in cell-selective drug delivery systems because they increase nanoparticle-cell interactions and uptake of carried drugs. Currently, the potential of NP glycosylation to enhance the safety and efficacy of carried therapeutic antitumor agents has been widely acknowledged, and much information is accumulating in this field. This review seeks to highlight recent advances in NP stabilization, toxicity reduction, and pharmacokinetic improvement and the promising potential of NP glycosylation from the perspective of molecular mechanisms described for drug delivery systems for cancer therapy. From preclinical proof-of-concept to demonstration of therapeutic value in the clinic, the challenges and opportunities presented by glycosylated NPs, with a focus on their applicability in the development of nanodrugs, are discussed in this review.

## Introduction

Nanoparticles have long been known as the foremost systems to improve drug delivery for treatment of several diseases, especially cancer. However, development of effective, targeted, and safe drug delivery systems remains challenging in many cancer types due to limited target sites (1). Therefore, to develop strategies that facilitate specific delivery of therapeutic agents to the target site, reducing access to nontarget sites is urgently needed (2, 3). One strategy for applying targeted therapies is the use of carbohydrates and monosaccharides as ligands that represent crucial structures on tumor cell membranes and have been shown to be effective for cell-selective drug delivery (4).

Cancer metabolism is also a promising target for cancer therapy in the nanomedicine field. According to the classic theory known as “the Warburg effect,” cancer cells require a much higher glucose flux than normal cells because their phenotype is characterized by preferential dependence on glycolysis for energy production in an oxygen-independent manner (5). Hence, certain key proteins involved in this disruptive metabolism, such as GLUT, hexokinase-2 (HK2) and phosphoglycerate dehydrogenase (PHGDH), which are overexpressed in cancer, have been examined as possible targets (6). Additionally, energy source replacement with other monosaccharides, such as mannose, could retard tumor progression (7). Currently, repurposing of nanocarriers conjugated with glycan-based molecules is an interesting field of opportunity for cancer therapy and diagnosis. Hence, a wide range of functional nanocarriers, including polymeric, metallic, and metalorganic NPs, are being studied and developed in the biomedical field (8). NPs possess unique physical, optical, and electrical proprieties and can be conjugated with several therapeutic and target molecules that modify their interactions with cell membranes and biological systems, altering their toxicity and pharmacokinetic profiles (9). Furthermore, adsorption or conjugation of glycan structures can change the intrinsic properties and mobility of NPs in biological systems. Glycosylated nanomaterials interact differently with tumor-associated glycoprotein receptors, and generally, binding can be achieved through multivalent carbohydrates because both the membrane and microenvironment of cancer cells have been well studied (10–12). Therefore, this review aims to highlight the current novel strategies that have been developed for cancer therapy through the use of drug delivery systems that include carbohydrate-based NP systems as dendrimers, micelles, silica, and lipidic and metallic NPs, exploiting the modified metabolism of cancer cells as a therapeutic approach.

## Glucose Metabolism and Transporters in Cancer Cells

**Figure 1:**
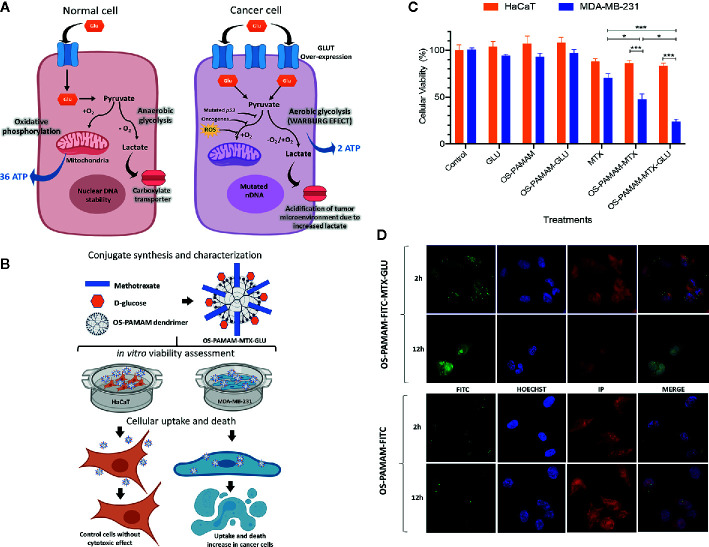
A graphical representation of Warburg effect in cancer and experimental demonstrations of the improvement of glycosylated drug delivery systems for target cancer therapy **(A)** Metabolic differences between normal and cancer cells. In the presence of O_2_, normal cells metabolize glucose in pyruvate followed by oxidative phosphorylation in the mitochondria generating 36 ATP per glucose molecule. In the deficiency of O_2_, pyruvate is transformed to lactate *via* anaerobic glycolysis generating 2 ATP per glucose molecule. In cancer cell, mutations in mtDNA, nDNA or absence of *p53* gene, presence of oncogenes and ROS suppress oxidative phosphorylation and enhances lactate production *via* glycolysis even in the presence of O_2_ (Warburg effect). **(B)** Glycosylated PAMAM dendrimers conjugated with methotrexate as a strategy for breast cancer target therapy. **(C)** Comparison of viability between MDA-MB-231 and HaCaT cell lines. Cells were exposed to OS-PAMAM-MTX-GLU and control treatments at the same concentration of free MTX and GLU was calculated in encapsulation assay for 4 h. Data represent mean ± SD (n = 16). Statistical analysis was performed by two-way ANOVA followed by *post hoc* Tukey’s multiple comparisons test. ***P < 0.001, *<0.02. **(D)** Confocal images of MDA-MB-231 cells incubated for 2 and 12 h with OS-PAMAM-FITC and OS-PAMAM-FITC-MTX-GLU. For each group, the images from left to right showed the fluorescence of FITC (green), Hoechst 33342 (blue), and PI (red) stains. Images were acquired at 63×. Data has been contributed and modified from Torres-Pérez (14).

The modified metabolism in cancer cells, which resorts to preferential use of glycolysis as the main energy source for ATP generation, promotes cancer cell growth, survival, proliferation, and long-term maintenance (13). The ATP production efficiency of glycolysis is much lower than that of oxidative phosphorylation, and cancer cells adapt to this disadvantage by increasing glucose uptake (**Figure 1A**) (5). Indeed, in the clinic, it has been reported that a high blood glucose level is associated with a poor prognosis in cancer patients (15, 16). Therefore, glucose plays an important role in cancer progression because it promotes cancer cell proliferation in a dose-dependent manner (17, 18).

Glucose is a hydrophilic molecule that must be transported and modified by specific proteins in the cell. Two classes of transporters are present in cells: the family of GLUT proteins and sodium-dependent glucose transporters (SGLTs) (19). These molecules are overexpressed in cancer cells; therefore, their inhibition can be a therapeutic strategy against cancer (20, 21). The use of compounds that suppress the growth of cancer cells through inhibition of glucose transporters has been widely explored in various types of cancer, including liver, colon, ovary, prostate, brain, and breast cancer (21–26). For example, in ovarian cancer cells, GLUT-1 and GLUT-3 protein levels are increased 6.5 and 4.1 times, respectively, and a GLUT-1/-3 inhibitor prevents cell growth, targets metabolic plasticity, and overcomes the cellular rescue mechanisms of cancer cells (22).

## Glycosylation Affects Cancer Cell Membranes and the Microenvironment

Cancer cells exhibit membranal structure changes *via* changes in external monosaccharide-related target molecules, such as proteins and lipids, that aid in tumorigenesis, malignant transformation, and tumor dissemination (27). For example, overexpression of sialic acid on the cell surface creates a negative charge on membranes and repulsion between cells, which helps cells enter the bloodstream (28). Changes in the intrinsic glycosylation of cell surface adhesion molecules, such as selectin ligands, integrins, and mucins, have been implicated in changes in the tumor microenvironment that can contribute to drug resistance and pH acidification (29), which lead to more aggressive cancer cell phenotypes; thus, their implications in the design of glycan-based therapies should be investigated (30). Therefore, glycans, glycoproteins, glycan-binding proteins, and proteoglycans are mechanistically implicated in cancer hallmarks (31, 32). For instance, lowered tumor extracellular pH (pHe) and upregulation of the membrane protein matrix metalloproteinase 2 (MMP2) in the tumor microenvironment has been exploited as a strategy to improve the selectivity of plasmid DNA release. Hence, DendriGraft poly-lysine, third-generation, (DGL-G3) conjugated with a cell-penetrating peptide (CPP), quenched by a pH-sensitive masking peptide, and linked by a metalloproteinase MMP2 substrate was a successful gene delivery system in a hepatoma cell line (32, 33).

Furthermore, tumor-associated macrophages (TAMs) can remodel the tumor microenvironment to reduce growth barriers, such as the dense extracellular matrix, and shift tumors towards an immunosuppressive microenvironment that protects cancer cells from targeted immune responses, making it difficult to deliver drugs with NPs larger than 100 nm (34). Glycoconjugates, such as mesoporous silica NPs (MSNs), can interrupt these biological interactions within tumors by altering TAM phenotypes through a process called polarization. By treating these MSNs with deglycosylases, the surface glycosylation of these NPs can be modulated without altering the protein coating. Reports indicate that increasing the size of silica particles can reduce their cellular uptake and minimize their M1-like macrophage polarizing capability, and surface modification of MSNs can further control their cellular uptake and modulate their polarization effects (28, 34, 35). Therefore, further investigation is required to determine the complete effects of carbohydrate changes in the external microenvironment and their role in inhibition of tumorigenesis.

## Carbohydrate-Based Carrier Molecules for Cancer Therapy

Specificity is a crucial aspect of drug administration in treatments against cancer because nonspecific agents can damage healthy tissues, causing adverse effects in patients (36). Carbohydrate changes in the external microenvironment of cancer cells also provide specific targets for carrier-based drug delivery. Hence, these carriers must be composed of biocompatible and biodegradable materials, which should be well characterized and conjugated (37). Among these, nanomaterials have been well accepted as nontoxic and nonimmunogenic agents (38).

NPs based on carbohydrates or conjugated to them have been explored as vehicles for drug administration in cancer (39, 40). Indeed, a wide variety of polysaccharides have been used, including chitosan (41), cellulose (42), glycogen (40), chitin (43), and dextran (44, 45), among others. There are two special cases. The first is hyaluronic acid (HA), a natural polysaccharide used in gene therapy and as a based-drug carrier. HA has shown a high molecular interaction with the CD44 receptor protein, a cell-surface glycoprotein involved in cell-cell interactions that is overexpressed in several types of cancer cells (46, 47). The second is the chitosan NPs, which are self-assembled, low-cost nanostructures with high positive charges that have the ability to encapsulate and deliver hydrophobic and negatively charged drugs to cancer cells (48). Chitosan can be accumulated accurately by proving the interaction of charges and permeability with the cancer cell membrane. Also, it has shown a high biodegradability in sub-components of glutamic acid (49). This type of NP can be preferentially internalized *via* receptor-mediated endocytosis. Uptake studies have demonstrated an increase in the endocytic pathway, with both clathrin and caveolae activation, when receptors on the cellular membrane were blocked. Therefore, the intrinsic properties of NPs conjugated with ligand molecules, such as folic acid, can significantly improve drug delivery in chemotherapy strategies and reversion of multidrug resistance (50, 51).

## Conjugation Strategies for Glycosylated Nanoparticles Used in Cancer Therapy

Setting up a conjugation method requires several considerations, starting from an understanding of the chemical composition of both the cargo and the carrier molecule. The chemical composition of cargo molecules influences the physicochemical properties of nano systems including size, surface charge, and shape, but also, modifying biological effects. For therapeutic purposes, glycosylated nanoparticles (G-NPs) should be biocompatible, biodegradable, and soluble in biological fluids, and most importantly, they must have receptor-targeting properties (4, 8).

The most common monosaccharides, including glucose, mannose, fructose, and galactose, have usually been applied in the synthesis of glycoconjugates because of the ease of conjugation and their specific effect as a targeting ligand to some key receptors found in cancer cells (4). Monosaccharide molecules possess several groups, such as hydroxyl groups, which can be highly reactive to generate stable conjugation with carrier NPs through various linkage approaches, such as reductive amination (52, 53). Drug carriers usually have amino-terminal groups that allow hydroxyl groups to be linked directly to both NPs and/or drugs through the following strategies (54, 55):

1. Direct amide linkages with sugar-bearing carboxylated or activated ester derivatives. This is beneficial for conjugation of monosaccharides, for example, in surface-amino dendrimers modified with chemo drugs against breast cancer and glioma (14, 56), including antitumor immunotherapy using chitosan NPs and TCL vaccines coupled with mannose to target specific moieties in dendritic cells (DC) (57).
2. Introduction of thiourea linkages formed by treatment of NP-amino groups with isothiocyanate saccharide derivatives. This coupling is helpful for theragnostics when different linkage strategies must be employed for different cargo molecules or NP systems and has been used in dendrimers premodified with fluorescein isothiocyanate but also linked to gold NPs (58).
3. Monosaccharides can also be found in the derivate version containing amino groups, which are frequently used for carriers with peripheral carboxyl groups, for example, D-mannosamine conjugated to solid lipid nanoparticles (SLNs) through amidization. The resulting p-aminophenyl-a-D-mannopyranoside-modified SLNs (MAN-SLNs) effectively delivered docetaxel to the brain (59).

The advantages of these strategies include the following: i) the reactions are conducted at room temperature and are compatible with most drugs and degradable linkers; ii) the resulting products, such as poly(monochlorotriazine), can be conveniently derivatized (i.e. PEGylated). However, direct sacrifice of the reducing sugars, formers of extended linkers *via* amide-bond formation starting from sugar lactones described in the first syntheses, should be avoided, and the NP must have a spherical architecture to avoid a chelating effect (60).

## Physical Properties of Glycosylated Nanoparticles

The performance of drug delivery systems based on NPs in cancer therapy is affected by several physical properties, mainly size, shape, and surface electric charge, which modulate NP toxicity and stability. Also, these characteristics should be considered for glycoconjugates because most interactions with altered membrane molecules are closely related to the aforementioned parameters (61). In NPs, small changes in structure can lead to significant changes in properties and reactivity. Additionally, the directional organization of molecules on the nanoparticle periphery can help by increasing the electrophile affinity to target molecules due to the high surface area to volume ratio of NPs (62, 63). Therefore, the optimum drug dispersion and homogeneity in a nanoparticle system and the linkage to cargo molecules should be well controlled and reproducible to obtain the desired therapeutic effect (64).

Regarding size, reports on organic and inorganic NPs indicate that glycosylation increases the size and molecular weight of NPs (14, 64, 65). Additionally, glycoconjugates exhibit a neutralization of zeta potential without significant alterations in colloidal stability (34, 66). Furthermore, depending on the drug conjugation approach and the therapeutic strategy, cationic saccharide molecules, such as dextran spermine and aminated pullulan, or anionic molecules, such as pectin, heparin, and hyaluronic acid, can be modulated to obtain the desired therapeutic effect (67).

Regarding cancer therapy with drugs, it is crucial to avoid side effects due to the toxicity of NPs. Nonspecific toxicity is primarily influenced by surface chemistry, functionality, size, chemical composition, and zeta potential (65, 68). Organic glycoconjugates are natural products of living systems also upshot as multifaceted drug delivery vehicles that can reduce the toxicity associated with unmodified drug carriers and therapeutic agents. An additional attribute of these carriers is their ability to positively alter the pharmacokinetic profile of drugs through stabilization (2, 38, 69). Furthermore, glycans and carbohydrates can neutralize the very positive or very negative charges of NPs, such as dendrimers or gold NPs, which can compromise the integrity of the plasma membrane, causing necrotic cell death (70, 71). Therefore, attached glycans play a critical role in maintaining NP stability and conformation and can define many of the physical properties of NP systems, which positively influences the safety of the proposed nanosystems through improvement of pharmacokinetic and biocompatibility (35, 72, 73).

## Applications of Glycan-Based Nanoparticles

Glycan changes in malignant cells, a hallmark of cancer, take a variety of forms: increase in incomplete or truncated glycan expression, loss of expression or excessive expression of certain glycans, and, less frequently, the appearance of novel glycans (26, 74). Furthermore, G-NPs have been studied to improve specific delivery of known and reassigned drugs as well as DNA, proteins, and peptides like vaccines. A database search was carried out with the words “glycoconjugates,” “glycopolymers,” “glycodendrimers,” and “glycol AND drugs” “glycosylation AND nanoparticles AND cancer” in the Scopus server and Integrity (https://integrity.clarivate.com/integrity/xmlxsl/). The search revealed the increasing amount of research on G-NPs during the last 20 years (approximately 3,500 patents), especially because the number of technology patents around the world has doubled in the last 10 years. Therefore, these types of nanosystems have the potential to be used in cancer therapy and prevention, pathological imaging diagnosis, and theragnostics.

### Glycosylated Nanoparticles as Carriers of Drugs and Small Molecules

The most common strategies for cancer therapy include the use of small molecular drugs, and NP systems improve the pharmacokinetic and pharmacodynamic profiles of these drugs due to the ability of NPs to remain in prolonged circulation in systemic models, increasing drug biodistribution and circulation, and reducing *in vivo* side effects (75, 76). For instance, overexpression of GLUT in breast cancer cells can enhance drug uptake (77). Moreover, our group performed a therapeutic strategy that included glycosylation of a one-step PAMAM dendrimer loaded with methotrexate (OS-PAMAM-MTX-GLU) (**Figure 1B**). This study showed that glucose conjugation led to a 150% increase in the internalization of OS-PAMAM conjugates in MDA-MB-231 breast cancer cells and reduced cell viability by up to 20%. Cancer cell death was significantly higher with the nanosystem than with free MTX, and the system displayed specificity because no effects were observed in noncancer cells (**Figures 1C, D**) (14).

Gold glyconanoparticles coupled to listeriolysin O 91–99 peptide (GNP-LLO_91–99_) have been used as a novel adjuvant for cancer therapy. GNP-LLO_91–99_ exhibited antitumor activity by inhibiting tumor growth and migration in melanoma cells and generated an immune response by recruiting and activating DC (78). In addition, other strategies, including two glycosylated systems to deliver cisplatin (CDDP), mannose-decorated tobacco mosaic virus (CDDP@TMV-Man) and lactose-decorated tobacco mosaic virus (CDDP@TMV-Lac), have been reported. CDDP@TMV-Man induced enhanced endocytosis and apoptosis in galectin-rich MCF-7 cells, whereas CDDP@TMV-Lac showed superiority in endocytosis and apoptosis in HepG2 cells with overexpression of asialoglycoprotein receptors (ASGPR) (79). Currently, other strategies for cancer drug delivery using glycosylated carriers have shown a high antitumoral effect, reaching up to 95% cell death. In particular, the high affinity of galactose for the asialoglycoprotein receptor in cancer cells has provided outstanding therapeutic strategies, with special benefits in liver cancer (**Table 1**).

**Table 1:** Applications of the recent glycosylated nanoparticles for drug delivery in cancer cells.

Carrier	Average size ± SD (nm)	Ligand	Receptor	Applications	Cell line/cancer model	The decrease in tumor volume/cell viability (%)	Decrease in control cells (%)	Reference
Glycogen nanoparticles	175 ± 75	Galactose	Asialoglycoprotein receptor	The system has efficient accumulation and release of drugs at tumor sites, inhibiting tumor growth with only slight retention in normal liver tissues.	*In vivo* model HepG2/Liver epithelial cells	80	15	(40)
Solid-lipid nanoparticles	174,51 ± 5.1	Fucose	Lectin receptors	Efficient delivery of methotrexate mediated by fucose-decorated solid lipid nanocarriers in breast cancer therapy.	*In vivo* model MCF7/Breast epithelial cells	75	-*	(75)
Liposomes	81.9 ± 6.2	Mannose-6-phosphate	Type II insulin-like growth factor receptor	Selective induction of apoptosis in MCF7 cancer cells by specific liposomes functionalized with mannose-6-phosphate.	*In vitro* model MCF7/Breast epithelial cells	50	No significant differences with untreated cells	(80)
Polymer nanoparticles	54,84 ± 0.58	Galactose	Asialoglycoprotein receptor	Galactose-Containing Polymer-DOX Conjugates for Targeting Drug Delivery.	*In vitro* model HepG2/Liver epithelial cells	80	55	(81)
Polyethyleneimine-modified iron oxide nanoparticles	98.2 ± 2.3	Galactose	Asialoglycoprotein receptor	Targeted delivery and accumulation of siRNA in tumor cells for therapy of hepatocellular carcinoma.	*In vivo* model Hepa 1–6/Liver epithelial cells	70	–	(82)
Polymer nanoparticles	112 ± 5	Galactose	Asialoglycoprotein receptor	Polymeric NPs as potential carriers for hepatoma‐targeted drug delivery and liver cancer therapy in clinical medicine.	*In vitro* model HepG2/Liver epithelial cells	95	No significant differences with untreated cells	(83)
Lipid nanoparticles	228,8 ± 5.42	Mannose	Mannose receptor	Increased supply of gemcitabine in lung cancer cells. The mannosylated formulation has higher cytotoxicity and can selectively kill cancer cells.	*In vitro* model A549/Lung epithelial cells	35	–	(84)
Lipid nanoparticles	239 ± 2,4	Galactose	Lectin receptors	Targeted delivery of doxorubicin to lung cells induces increased cytotoxicity related to that related to marked drug uptake and accumulation.	*In vitro* model A549/Lung epithelial cells	30	–	(85)
Mesoporous silica nanoparticles	180 ± 50	Mannose	Lectin receptors	Nanoparticles conjugated with D-mannose vehicles for controlled drug release in A549 cells.	*In vitro* model A549/Lung epithelial cells	45	10	(86)

*Not determined.

### G-NP Carriers of Nucleic Acids

Due to recent developments in gene therapy, G-NPs have been employed for specific and higher nucleic acid (siRNA, DNA, and miRNA) transfection. A series of cationic block copolymers (PHML-*b*-PMAGal) and the statistical copolymers P(HML-*st*-MAGal) with pendant natural galactose and (L-)-lysine moieties were exposed to a human non-small cell lung carcinoma cell line. P(HML_40_-*st*-MAGal_4_) with 4.8% galactose content showed the highest gene transfection efficiency among the synthesized cationic polymers, 6.8-fold higher than the “gold standard” bPEI-25k (87). Combined treatments, such as using targeted NPs to deliver chemopeptides and gene therapeutics, have been delivered efficiently to cancer cells and tissues to avoid transfection cytotoxicity, overcome drug resistance, and stop tumor development. In one study, a novel mannosylated copolymer with a CPP grafted into Polyethylenimine (PEI) was prepared to target antigen-presenting cells (APCs) with mannose receptors. The gene transfection was significantly higher by the grafted CPP mannosylated than in control cells (88, 89).

### G-NP Applications in Immunotherapy and Vaccines

The presence of altered glycans on cancer cells has been used as a diagnostic marker and tumor cell marker (90). Glycan aberrations have not only been used as markers but can also be linked to endogenous lectins, such as galectins, sialic acid-binding immunoglobulin type lectins, and selectins (91). For example, type C lectin receptors are widely expressed on myeloid cells, such as macrophages, neutrophils, and DC. Consequently, they can mediate specific interactions with tumor antigens and facilitate tumor rejection (92, 93).

Due to their relevance, incomplete or truncated glycan structures, often covered by sialic acid and commonly known as tumor-associated carbohydrate antigens (TACA), have been studied (94). These antigens have already been seen to be overexpressed in different cancer types, such as breast, pancreas, bladder, and colon cancer (95–98). For example, glycodendrimers were evaluated due to their dual properties as targeting agents using a CD4- and CD8-directed melanoma antigen (gp100) and a glycan (LeY) recognized by the type C lectin receptors DC-SIGN and Langerin. Thus, the first glycovaccine with dual C-type lectin receptors (CLR) targeting properties was designed with glycosylated dendrimers, which reached multiple human skin DC and improved antitumor CD8+ T cell responses (99). These investigations demonstrate that glycans can be applied both in the construction of systems to detect biomarkers for tumor diagnosis and prognosis determination, as well as in the development of vaccines targeting carbohydrate antigens (91).

### G-NPs Used in Theragnostics

The Warburg effect is a hallmark of cancer and serves as a target for both diagnosis and therapeutic strategies (100). Several glycoconjugates, such as 99mTc-labeled deoxyglucose derivates and glucosamine functionalized with multiwalled carbon nanotubes, have been employed as diagnostic agents for heart and brain cancer and showed superior accuracy over current diagnostic methods (101, 102). However, in recent years, theragnostic systems, such as silica and hyaluronic acid-based NPs that can be used to image cancer cells and at the same time can suppress tumor growth, have been designed by improving the solubility of hydrophobic drugs and glycosylation-mediated drugs and the tumor cell targeting efficiency, with minimum toxicity (103–105).

## Conclusions and Perspectives

Current evidence indicates that glycosylation strategies combined with drug delivery systems and immunological therapy present potential opportunities for cancer therapy and theragnostics. In particular, nanosystems proposed for lipidic NPs with galactose are the most well studied and promising strategy against several cancer types. However, targeted G-NPs for cancer treatment involving novel nanotechnologies and medical strategies have numerous challenges and issues. One of the challenges of targeted NPs is to induce a beneficial alteration in the solubility, stability, and pharmacokinetic features of the drug carried. Other challenges are related to control the diverse alterations in the tumoral microenvironment and the clinical safety and repeatability concerns.

Further nanomedicine innovations and basic research are crucial for the discovery of more specific cancer receptors and new glycan-based ligands or repurposed drugs against these receptors. Although the majority of carbohydrates and chemo drugs used in these experimental therapies are low-cost molecules, the sum of all the components and synthesis steps necessary to obtain the nanoconjugate can be expensive, and researchers have not fully examined the cost-effectiveness issues. Apart from accumulation of nonmetabolizable nanocomponents like gold, leakage of shelf life, toxicity of some substances employed for making NPs is another restriction. Therefore it is recommended to use organic NPs for therapeutic applications.

## Author Contributions

ST-P and ER-G designed this work of review. CT-P, ST-P and MP-E performed the literature search of the databases. ST-P and CT-P Writing—original draft preparation. ER-G, MP-E and SP-T Supervision, writing—reviewing and editing. All authors contributed to the article and approved the submitted version.

## Funding

This project was financed by Secretaría de Investigación y Posgrado (SIP-IPN) through project 2020205. Consejo Nacional de Ciencia y Tecnología (CONACyT) through Fondo Sectorial de Investigación para la Educación through project No. A1-S-21548 and Fondo de Investigación Científica y Desarrollo Tecnológico through Instituto Politécnico Nacional.

## Conflict of Interest

The authors declare that the research was conducted in the absence of any commercial or financial relationships that could be construed as a potential conflict of interest.
